# PARP14 is an interferon-induced host factor that promotes IFN production and affects the replication of multiple viruses

**DOI:** 10.1128/mbio.02299-25

**Published:** 2025-09-12

**Authors:** Srivatsan Parthasarathy, Pradtahna Saenjamsai, Hongping Hao, Anna Ferkul, Jessica J. Pfannenstiel, Daniel S. Bejan, Yating Chen, Ellen L. Suder, Nancy Schwarting, Masanori Aikawa, Elke Muhlberger, Adam J. Hume, Robin C. Orozco, Christopher S. Sullivan, Michael S. Cohen, David J. Davido, Anthony R. Fehr

**Affiliations:** 1 Department of Molecular Biosciences, University of Kansas 4202, Lawrence, Kansas, USA; 2 Department of Chemical Physiology and Biochemistry, Oregon Health Sciences University 6684, Portland, Oregon, USA; 3 Department of Molecular Biosciences, University of Texas 12330, Austin, Texas, USA; 4 Department of Microbiology, Boston University School of Medicine 12259 , Boston, Massachusetts, USA; 5 National Emerging Infectious Diseases Laboratories, Boston University 1846, Boston, Massachusetts, USA; 6 Center for Emerging Infectious Diseases Policy & Research, Boston University 1846, Boston, Massachusetts, USA; 7 Center for Excellence in Vascular Biology (P.K.J.E.A.), Brigham and Women’s Hospital, Harvard Medical School 1811 , Boston, Massachusetts, USA; 8 Center for Interdisciplinary Cardiovascular Sciences (M.A.E.A.), Brigham and Women’s Hospital, Harvard Medical School 1811 , Boston, Massachusetts, USA; 9 Channing Division of Network Medicine (M.A.), Brigham and Women’s Hospital, Harvard Medical School 1811 , Boston, Massachusetts, USA; Duke University School of Medicine, Durham, North Carolina, USA

**Keywords:** ADP-ribosylation, PARP, macrodomain, coronavirus, HSV-1, VSV, LCMV, interferon, interferon-stimulated genes

## Abstract

PARP14 is a 203 kDa multi-domain protein that is primarily known as an ADP-ribosyltransferase and is involved in a variety of cellular functions, including DNA damage, microglial activation, inflammation, and cancer progression. In addition, PARP14 is upregulated by interferon (IFN), indicating a role in the antiviral response. Furthermore, PARP14 has evolved under positive selection, again indicating that it is involved in host-pathogen conflict. We found that PARP14 is required for increased IFN-I production in response to coronavirus infection lacking ADP-ribosylhydrolase (ARH) activity and poly(I:C); however, whether it has a direct antiviral function remains unclear. Here, we demonstrate that the catalytic activity of PARP14 enhances IFN-β and IFN-γ responses and independently restricts ARH-deficient murine hepatitis virus (MHV) and severe acute respiratory syndrome coronavirus 2 (SARS-CoV-2) replication. To determine if PARP14’s antiviral functions extended beyond CoVs, we tested the ability of herpes simplex virus 1 (HSV-1), a DNA virus, vesicular stomatitis virus (VSV), a negative-sense RNA virus, and lymphocytic choriomeningitis virus (LCMV), an ambisense RNA virus, to infect A549 PARP14 knockout (KO) cells. While LCMV infection was unaffected, HSV-1 replication was increased in PARP14 KO cells, and VSV replication was decreased. These results indicate that PARP14 restricts HSV-1 replication but enhances the replication of VSV. A PARP14 active site inhibitor had no impact on HSV-1 or VSV replication, indicating that its effect on these viruses was independent of its catalytic activity. These data demonstrate that PARP14 promotes IFN production and has both proviral and antiviral functions targeting multiple viruses.

**IMPORTANCE:**

The antiviral response is largely regulated by post-translational modifications (PTM), including ADP-ribosylation. PARP14 is an ADP-ribosyltransferase that is upregulated by interferon and is under positive selection, indicating that it is involved in host-pathogen conflict. However, no anti-viral function has been described for PARP14. Here, we found that PARP14 represses both coronavirus and herpes simplex virus 1 (HSV-1) replication, demonstrating that PARP14 has antiviral functions. Surprisingly, we also found that PARP14 has pro-viral functions, as it was critical for the efficient replication of vesicular stomatitis virus (VSV). These data indicate that PARP14 has both proviral and antiviral functions. Defining the mechanisms used by PARP14 to both repress and promote virus replication will provide new insights into how PARPs regulate virus infection.

## INTRODUCTION

Virus infection causes activation of host immune responses, with the interferon (IFN) response being the major pathway activated. Recognition of viral pathogen-associated molecular patterns by host surface and cytosolic receptors leads to a series of molecular events that activate the expression of different types of IFNs, which eventually upregulate the expression of anti-viral IFN-stimulated genes (ISGs). Several different post-translational modifications regulate this response, including phosphorylation, ubiquitination, and other less common modifications such as ADP-ribosylation.

ADP-ribosylation is a post-translational modification (PTM) where ADP-ribose subunits are added to target proteins in a process catalyzed by ADP-ribosyltransferases (ARTs) that use NAD^+^ as a substrate. The ART Diphtheria toxin-like domain (ARTD) family of ART proteins are intracellular ARTs that are individually named PARPs (1). These ARTDs catalyze the addition of either poly-ADP-ribose (PAR) or mono-ADP-ribose (MAR) to proteins or nucleic acids. The human genome encodes 17 PARPs that are involved in the regulation of most cellular activities, including DNA damage repair, ER stress, cell cycle regulation, transcription and translation, and the innate and adaptive immune responses (1). Apart from the addition of ADP-ribose, the removal of ADP-ribose from target proteins is also implicated in important cellular processes. The presence of ADP-ribose hydrolases (ARH) or macrodomains such as PARG, TARG1, MacroD1, and MacroD2 in humans underscores the importance of ADP-ribose turnover (2). Furthermore, all coronaviruses (CoVs) encode for a highly conserved macrodomain (Mac1) that removes ADP-ribose from proteins, indicating that these viruses may be susceptible to antiviral ADP-ribosylation. Infection with murine hepatitis virus (MHV), severe acute respiratory syndrome coronavirus (SARS-CoV), or SARS-CoV-2 containing point mutants in key residues required for catalytic activity (i.e., MHV N1347A) or full deletions of Mac1 induce robust interferon responses and are highly attenuated *in vivo* (3–5). In addition, the poor replication and enhanced induction of IFN following infection with MHV N1347A could be reversed using PARP inhibitors (3). These results demonstrated that PARP-mediated ADP-ribosylation is a potent anti-viral defense mechanism against CoVs.

The expression of several PARPs is activated by IFN in response to virus infection, categorizing them as ISGs. Some PARPs have known antiviral activity, including PARP13 (zinc-antiviral protein or ZAP), PARP12, PARP11, PARP9, PARP5a/b, PARP7, and PARP1 (6). In addition, some PARPs also act as pro-viral factors, promoting viral replication and infection. PARP11 was shown to suppress IFN-I signaling by MARylating IFNAR and priming it for ubiquitination and eventual degradation. The suppression of IFN-I signaling by PARP11 increased the replication of vesicular stomatitis virus (VSV) and herpes simplex virus 1 (HSV-1). These results demonstrated a pro-viral role for PARP11 (7). PARP7-dependent ADP-ribosylation also suppressed IFN-I production and promoted influenza A virus infection, indicating that PARP7 also has pro-viral functions (8). Consistent with this pro-viral function, PARP7 knockdown reduced the replication of MHV A59, demonstrating its ability to promote coronavirus replication (3). Finally, PARP1 inhibitors have also been shown to efficiently inhibit replication of several viruses such as herpesviruses, adenoviruses, and human immunodeficiency virus, indicating that PARP1 may also promote virus replication in some cases (9–11).

PARP14 is an ISG that is important in several different biological processes, including inflammation, cancer progression, and DNA damage. PARP14 is a multidomain protein, and its domain architecture was recently re-analyzed using Alphafold2 (12). This analysis determined that PARP14 contains three RRM domains, seven full KH domains, and one split KH domain that are suspected to bind to nucleic acids, three macrodomains (MDs), one of which has an ADP-ribose hydrolase activity (MD1) (12–14), a WWE domain that is suspected to bind ADP-ribose subunits, and the ART catalytic domain (12).

PARP14 was originally identified as a STAT6-binding protein that promoted IL-4-dependent anti-inflammatory signaling (15). Since then, several independent studies have implicated PARP14 in inflammatory and interferon signaling pathways. PARP14’s involvement in inflammatory and IFN responses is cell-type and insult dependent, suggesting that PARP14 has multiple functions that are context dependent. In M1 macrophages, PARP14 suppresses IFNγ-induced activation of pro-inflammatory signaling by STAT1 MARylation (16). We and others have found that PARP14 is also critical for inducing high levels of IFN-β following infection with *Salmonella typhimurium*, MHV, as well as treatments with lipopolysaccharide (LPS) or poly(I:C) (3, 17). Specifically, PARP14 knockout M0 macrophages and epithelial cells demonstrated significantly reduced levels of IFN-β after infection or treatment with LPS or poly(I:C), indicating that one of PARP14’s functions is to promote IFN-β production; however, the mechanisms by which it enhances IFN-β production remain unknown (3, 17). More recently, several groups have demonstrated that PARP14 is the major PARP responsible for IFN-γ and poly(I:C) induced cytosolic ADP-ribose bodies (ICAB) (18–21).

Interestingly, PARP14 is one of the several PARPs that have evolved under positive selection, indicating that it may be involved in host-pathogen conflicts. This positive selection is seen in many of the different domains of PARP14, especially the macrodomains, WWE domain, and the catalytic domain at the C-terminus of the protein (22). However, despite this evidence for involvement in host-pathogen interactions, there are very few reports describing any impact of PARP14 on virus or bacterial infections. In one report, PARP14 knockout (KO) RAW264.7 macrophages had significantly increased levels of *S. typhimurium* replication (17). In addition, siRNA knockdown of PARP14 led to a mild increase (approximately twofold) in the levels of a Mac1 mutant MHV viral RNA, but knockdown or a congenital knockout of PARP14 did not significantly increase the amount of infectious virus produced (3). Despite PARP14 being a regulator of IFN production and triggering the production of ICABs, it remains unclear how or even if PARP14 impacts virus replication.

Recently, several tools have been developed to facilitate testing the impact of PARP14 on virus infection. First, we reported PARP14 KO A549 and NHDF cell lines (3), which have intact IFN systems and are susceptible to infection by a wide variety of viruses. Second, a highly potent and selective PARP14 catalytic inhibitor, RBN012759, was developed (23). Finally, we report here the creation of a tamoxifen-inducible PARP14 knockout mouse model. Hence, with the availability of an advanced PARP14 inhibitor and with the advent of several PARP14 knock-out systems, we sought to determine the effect of PARP14 and its catalytic activity on IFN production and the replication of multiple different virus families.

Utilizing these tools, we report here that PARP14 influences the replication of multiple viruses. PARP14 potently inhibited the replication of HSV-1, as well as Mac1-mutant MHV and SARS-CoV-2. Unexpectedly, PARP14 also acted as a proviral factor for VSV, a negative-sense RNA virus, highlighting the multifaceted effect of PARP14 on virus replication. Furthermore, we confirmed that the catalytic activity of PARP14 was critical for IFN-β and IFN-λ induction and the inhibition of CoV replication. This study highlights the involvement of PARP14 in the IFN response and virus infection, underscoring the importance of PARP14 as an immunomodulatory host factor that can affect the replication of multiple viruses.

## RESULTS

### PARP14 promotes IFN-β and IFN-λ production in poly(I:C) transfected cells

**Fig 1:**
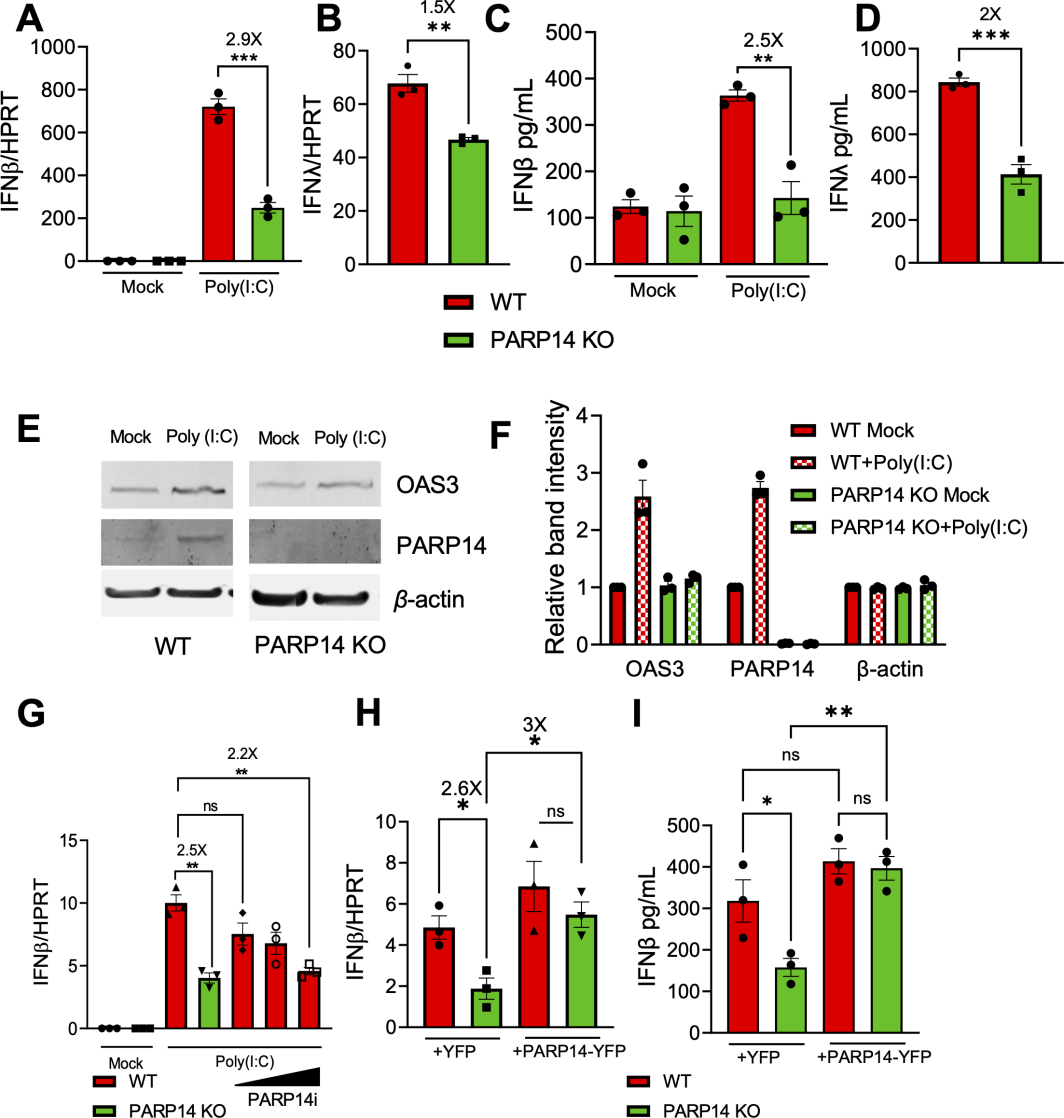
PARP14 promotes IFN production following poly(I:C) treatment in human A549 cells. (**A, B**) WT (red bars) and PARP14 knock-out (KO) (green bars) A549 cells were transfected with 0.5 ug/mL poly(I:C), RNA was isolated from cells at 18 hours post-transfection (hpt), and IFN-β (**A**) and IFN-λ (**B**) mRNA levels were quantified by qPCR using the ΔCt method. (**C, D**) WT and PARP14 KO cells were transfected with 0.5 µg/mL poly(I:C). Supernatant was collected 18 hpt, and IFN-β (**C**) and IFN-λ (**D**) protein levels were quantified by ELISA. Data shown in (**A–D**) are from one experiment and are representative of three independent experiments with N = 3 biological replicates for each experiment. (**E**) WT and PARP14 KO cells were either mock-transfected or transfected with 0.5 µg/mL of poly(I:C). Cell lysates were collected at 18 hpt, and OAS3 and PARP14 protein levels were determined by immunoblotting using β-actin as a loading control. (**F**) Band intensities from (**E**) were quantified by densitometry, normalized to β-actin, and then relative levels compared to mock-transfected WT cells were determined. Data shown in (**E, F**) are from one experiment and are representative of three independent experiments with N = 1 biological replicate per group in each experiment. (**G**) WT cells were either mock-transfected or transfected with 0.5 µg/mL of poly(I:C) and were immediately either treated with DMSO (vehicle) or with increasing concentrations of PARP14i (0.01, 0.1, and 1 µM). At 18 hpt, RNA was isolated from cells, and IFN-β mRNA was quantified by qPCR using the ΔCt method. (**H**) WT and PARP14 KO cells were transfected with 0.5 µg of YFP or YFP-PARP14 plasmids. At 48 hpt, cells were transfected with 0.5 µg/mL of poly(I:C), and 18 hours later, RNA was isolated from cells, and IFNβ mRNA was quantified by qPCR using the ΔCt method. (**I**) WT and PARP14 KO cells were transfected with 0.5 µg of YFP or YFP-PARP14 plasmids. At 48 hpt, cells were transfected with 0.5 µg/mL of poly(I:C) and 18 hours later, cell supernatant was used to quantify IFN-β protein by ELISA. Data shown in (**G–I**) are from one experiment and are representative of 2–3 independent experiments with *N* = 3 biological replicates per group for each experiment. Data in (**A**), (**B**), (**D**), and (**I**) were tested for statistical significance using a standard *t*-test and data in (**C**), (**G, H**) were tested for statistical significance using one-way analysis of variance (ANOVA).

We previously demonstrated that PARP14 was required for IFN-β production in response to poly(I:C) in human A549 cells via an indirect VSV bioassay (3). To determine how PARP14 impacts IFN induction more quantitatively, we induced IFN production via poly(I:C) transfection and quantified the amount of IFN-β and IFN-λ transcripts by quantitative PCR (qPCR) and protein by enzyme-linked immunosorbent assay (ELISA) in A549 wild-type (WT) and PARP14 KO cells, which were developed using CRISPR by transducing A549 cells with control or PARP14 targeting guide RNAs and Cas9 (3). Poly(I:C) transfection induced IFN-β (Fig. 1A; Fig. S1A) and IFN-λ (Fig. 1B) mRNA production in A549 cells, but their levels were significantly reduced in PARP14 KO compared to WT A549 cells. We also observed a similar decrease in IFN-β and IFN-λ protein levels following poly(I:C) transfection in PARP14 KO A549 cells compared to WT cells in an ELISA assay (Fig. 1C and D). Poly(I:C) transfected PARP14 KO cells also had decreased protein levels of the interferon-stimulated gene OAS3 compared to WT cells (Fig. 1E and F). Finally, we also observed a nearly fourfold reduction in IFN-β mRNA production in response to poly(I:C) transfection in PARP14 KO NHDF cells, indicating that the effect of PARP14 on IFN-β mRNA production in response to poly(I:C) is not limited to one cell type (Fig. S1B) (3). Since PARP14 catalyzes MARylation, we used a highly potent and selective PARP14 catalytic inhibitor, RBN012759 (PARP14i), to test if the observed reduction in IFN and ISG levels was due to the MARylating activity of PARP14. PARP14i efficiently restricted PARP14’s auto-MARylation activity in PARP14-overexpressing 293T cells in a dose-dependent manner (Fig. S1C). We utilized PARP14 G832E for these experiments as it was shown to be deficient in its MD1-dependent ADP-ribosyl hydrolase activity, which leads to enhanced autoMARylation (14). Importantly, poly(I:C) stimulated A549 cells displayed a dose-dependent decrease in IFN-β transcript levels when treated with PARP14i. At the highest concentration of PARP14i, the level of IFN-β mirrored that in PARP14 KO cells (Fig. 1G). This suggests that the reduction in IFN-β levels in PARP14 KO cells was due to the absence of PARP14 catalytic activity. In addition, we also used a PARP14-degrading compound, RBN012811 (DEG), and its associated negative control, RBN013527 (CTL), to test the role of PARP14 in IFN-β production (a generous gift from Mario Niepel, Ribon Therapeutics) (24). DEG effectively reduced PARP14 protein to undetectable levels in WT A549 cells (Fig. S1D). Compared to CTL, we found that the PARP14 DEG decreased IFN-β mRNA induction threefold, similar to results with complete PARP14 KO cells (Fig. S1E). Finally, we exogenously expressed a full-length PARP14 construct as a YFP fusion protein in PARP14 KO cells to demonstrate that PARP14 enhances IFN responses to poly(I:C) (Fig. S1G). Expression of YFP-PARP14, but not YFP, restored IFN-β transcript and protein abundance to near WT levels (Fig. 1H and I), further confirming that the decrease in IFN-β levels in PARP14 KO cells was not due to off-target effects of the PARP14 deletion. These results demonstrate that in response to poly(I:C) stimulation, PARP14-dependent MARylation promotes IFN-β and IFN-λ production.

### PARP14 promotes IFN-β production in Mac1-mutant CoV-infected cells

**Fig 2:**
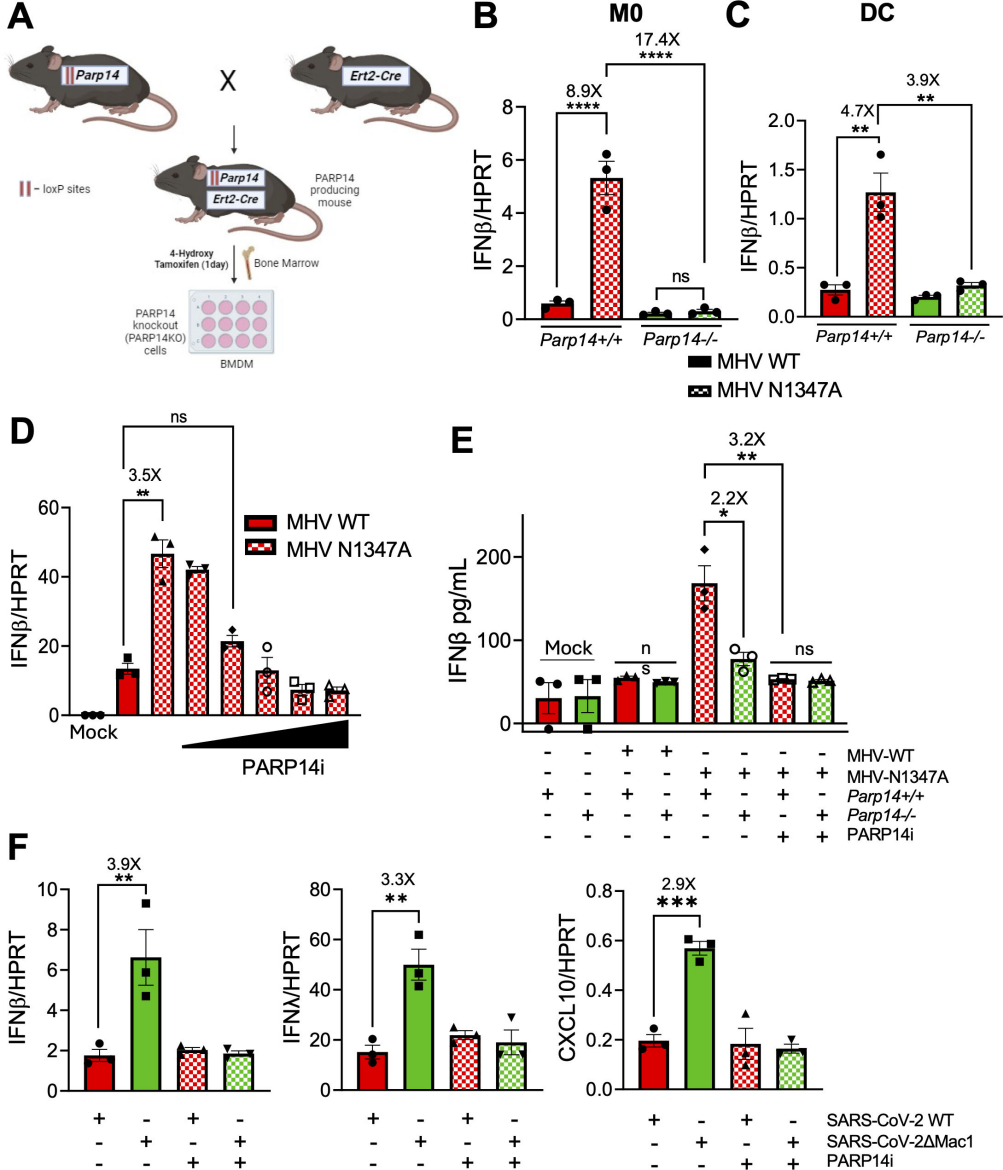
PARP14 promotes IFN production following Mac1 mutant CoV infection. (**A**) Schematic diagram showing the generation of PARP14 KO mice. *Parp14* floxed mice were crossed with *cre* heterozygote mice, and the resulting progeny were used to harvest bone marrow. Bone marrow cells were isolated and plated with 10 ng/mL of mCSF or GM-CSF for 6 days to differentiate cells into M0 macrophages or dendritic cells (DCs), respectively. On day 6, these cells were treated with 1 µg/mL of 4-hydroxy tamoxifen (4-OHT) for 24 hours to induce *Cre* expression, leading to the removal of exon 2 of *Parp14* (for details see Materials and Methods). (**B, C**) *Parp14^+/+^* (Cre-) (red bars) and *Parp14^−/−^* (Cre+) (green bars) M0 cells (**B**) or DCs (**C**) were infected with MHV WT (solid bars) or N1347A (checkered bars) at an MOI of 0.1. RNA was isolated from cells at 12 hpi, and IFN-β mRNA was quantified by qPCR using the ΔCt method. (**D**) *Parp14^+/+^* cells were infected with WT and N1347A at an MOI of 0.1 and then treated at 1 hpi with DMSO or increasing concentrations of PARP14 inhibitor (PARP14i) (3, 11, 33, 99, and 297 nM). RNA was isolated from cells at 12 hpi, and IFN-β mRNA levels were quantified using qPCR using the ΔCt method. (**E**) *Parp14^+/+^* and *Parp14^−/−^* M0 macrophages were infected with WT and N1347A MHV at an MOI of 0.1 and then treated with DMSO or 100 nM PARP14i at 1 hpi. The cell supernatant was collected at 12 hpi, and IFN-β protein was quantified by ELISA. (**F**) A549-ACE2 cells were infected with WT and ΔMac1 SARS-CoV-2 at an MOI of 0.1 and treated with DMSO or PARP14i. RNA was isolated from cells at 48 hpi, and IFN-β, IFN-λ, and CXCL-10 mRNA levels were quantified by qPCR using the ΔCt method. Data shown in (**B–F**) are from one experiment and are representative of three independent experiments with N = 3 biological replicates per group for each experiment. Data in (**B–F)** were tested for statistical significance using a one-way ANOVA.

Using congenital PARP14 KO mouse cells, we previously showed that PARP14 was required to promote IFN-β production upon infection with MHV-N1347A (3). To circumvent the limitations of constitutive PARP14 KO mice, which include poor breeding efficiency and potential confounding effects of developmental compensation for PARP14 KO by other PARPs, we developed a conditional tamoxifen-inducible PARP14 KO mouse model to better identify the role of PARP14 in response to MHV infection (Fig. 2A). *LoxP* sites were inserted into the *parp14* locus around exon 2 via CRISPR-mediated mutagenesis to create *parp14* floxed alleles. These mice were then bred with *ert2-cre* heterozygote mice to obtain both *parp14* floxed *ert2-cre* positive mice (*parp14^flox/Ert2-Cre^*), and as controls, *parp14* floxed ert2-*cre* negative offspring (*parp14^+/+^*) (Fig. 2A). Bone-marrow-derived macrophages (BMDMs) were harvested from these mice, and in *cre* positive cells, induction with 4-hydroxy tamoxifen (4-OH-T) led to an efficient knock-out of PARP14 (*parp14^−/−^*) (Fig. S2A). BMDMs that were *cre*-negative still expressed PARP14 following treatment with 4-OH-T (*parp14^+/+^*). Next, we tested the induction of IFN-β in both *parp14^+/+^* and *parp14^−/−^* BMDMs and dendritic cells (+ ), which were differentiated as described in Materials and Methods and confirmed by CXCR1 expression (Fig. S2B). MHV-N1347A infection induced robust IFN-β levels compared to WT virus in *parp14^+/+^* BMDMs and DCs (Fig. 2B and C) (3). However, in *parp14^−/−^* BMDMs and DCs infected with N1347A, the level of IFN-β was reduced to the same level as *parp14^+/+^* cells infected with WT virus, suggesting that PARP14 was required for IFN-β production in response to MHV-N1347A infection, confirming our prior observation and validating our KO mice (Fig. 2B and C). Similarly, there was also a decrease in the induction of ISG15 transcript levels in MHV-N1347A-infected cells in *parp14^−/−^* cells (Fig. S2C). To determine if PARP14’s ART activity was responsible for the increase in IFN-β expression following infection, MHV-WT and N1347A infected BMDMs were treated with PARP14i and tested for IFN-β mRNA expression. PARP14i treatment caused a dose-dependent decrease in IFN-β expression in BMDMs infected with N1347A (Fig. 2D), similar to its impact on poly(I:C) induced IFNβ expression. Following MHV-N1347A infection, the level of secreted IFN-β protein was also reduced to WT levels in *Parp14^−/−^* BMDMs and in WT BMDMs treated with PARP14i (Fig. 2E). Previously, we demonstrated that infection with a Mac1 deleted SARS-CoV-2 virus (ΔMac1) induced elevated levels of IFN-β and IFN-λ in human epithelial cells, which we hypothesized was due to PARP14 as it was highly upregulated following infection, though this was not formally proven (4). Following SARS-CoV-2 ΔMac1 infection of A549-ACE2 cells, PARP14i treatment of A549-ACE2 cells reduced the mRNA levels of IFN-β, IFN-λ, and CXCL-10 to levels seen following WT virus infection (Fig. 2F). These data demonstrate that the increase in IFN-β levels in response to Mac1-deficient human and mouse CoV infection occurs via PARP14-dependent MARylation.

### PARP14 promotes MDA-5 but not RIG-I-dependent IFN production

**Fig 3:**
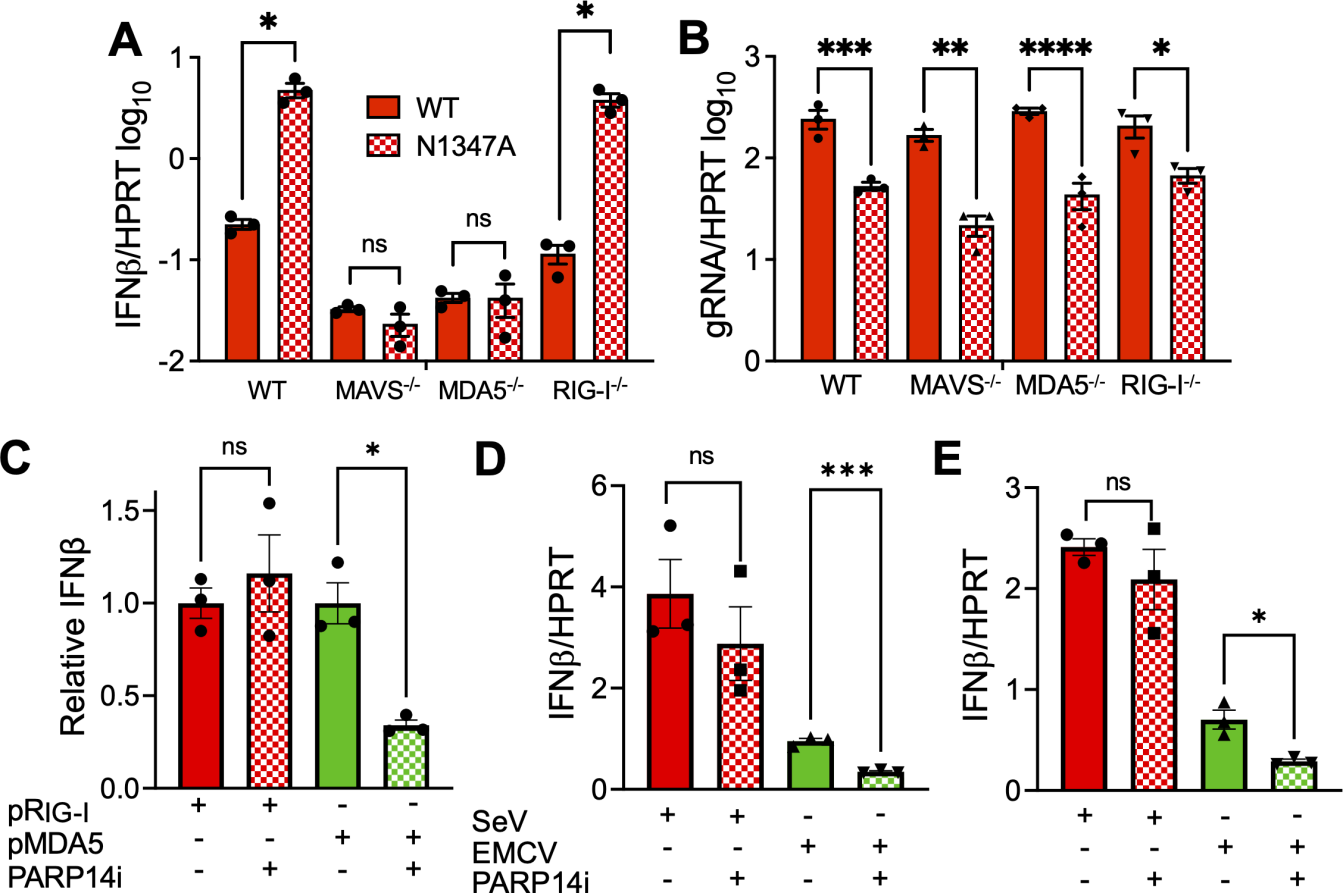
PARP14 promotes MDA-5-dependent IFN-β production. (**A**) BMDMs were generated from WT, MAVS^−/−^, MDA-5^−/−^, and RIG-I^−/−^ mice and infected with MHV WT or N1347A at an MOI of 0.1. RNA was isolated from cells at 12 hpi, and IFNβ mRNA was quantified by qPCR using the ΔCt method. (**B**) BMDMs were generated from WT, MAVS^−/−^, MDA-5^−/−^, and RIG-I^−/−^ mice and infected with MHV WT or N1347A at an MOI of 0.1. RNA was isolated from cells at 20 hpi, and MHV-JHM genomic RNA (gRNA) was quantified by qPCR using the ΔCt method. The results shown in (**A**) and (**B**) are from one experiment representative of two independent experiments, each with N = 3 biological replicates per group in each experiment. (**C**) A549 cells were transfected with 0.5 μg/mL of the indicated plasmid. Then either DMSO or 100 nM PARP14i was added to the cells 4 hpt. RNA was isolated from cells at 24 hpt, and IFNβ mRNA was quantified by qPCR using the ΔCt method. IFNβ transcript abundances were calculated relative to DMSO-treated control. (**D, E**) BMDMs (**D**) or A549 cells (**E**) were infected with either SeV at an MOI of 1 or EMCV at an MOI of 2. A 100 nM PARP14i or DMSO was then added at 1 hpi. RNA was isolated from cells at 24 hpi (A549 cells) or 36 hpi (BMDMs), and IFNβ mRNA was quantified by qPCR using the ΔCt method. The results in (**C–E**) are from one experiment representative of three independent experiments, each with N = 3 biological replicates per group for each experiment. All data in (**A**), (**B**), (**D**), and (**E**) were tested for statistical significance using a standard *t*-test between the indicated samples. Data in (**C**) were tested for statistical significance using a one-way ANOVA.

IFN stimulation in virus-infected cells occurs due to recognition of viral RNA by pattern recognition receptors such as MDA5 or RIG-I. This leads to a cascade of events, resulting in activation of cytoplasmic proteins such as MAVS, TBK-1, and IRF3, and the nuclear translocation of IRF3 that leads to transcriptional activation of IFN production. BMDMs infected with N1347A MHV-JHM induced a robust production of IFN-β compared to WT infection. However, this difference in IFN-β production was lost during N1347A infection in BMDMs from MAVS knock-out (KO) (MAVS^−/−^) mice, demonstrating the key role of intracellular RNA sensing for the induction of IFN-β (Fig. 3A). We then tested the effect of MDA5 and RIG-I KO (Mda5^−/−^ and RIG-I^−/−^) on IFN-β production following infection with N1347A (Fig. 3A). We found that RIG-I KO did not have any effect on IFN-β production during N1347A infection. In contrast, IFN-β levels were reduced to WT levels in Mda5^−/−^ BMDMs, suggesting the IFN-β production during N1347A infection occurred via the MDA5-dependent IFN-production pathway. As noted previously (3), this PARP14-dependent IFN production was independent of the repression of N1347A replication, as the N1347A replication defect, compared to WT virus, was seen in all KO BMDMs (Fig. 3B). Next, we tested whether PARP14 specifically regulated MDA5-dependent IFN-β production by overexpressing MDA5 or RIG-I in A549 cells, which is known to induce IFN expression. First, we confirmed that RIG-I and MDA5 were highly expressed following transfection and that expression of a control plasmid expressing GFP in the same backbone had little impact on the levels of IFN-β in our experiments (Fig. S3). IFN-β production due to exogenous expression of RIG-I remained unaffected following PARP14i treatment. However, there was a significant approximately threefold reduction in IFN-β production following PARP14i treatment in MDA5-expressing cells (Fig. 3C; Fig. S3). To corroborate these findings, we used two different viruses, Sendai virus (SeV) and encephalomyocarditis virus (EMCV), that are specific agonists of RIG-I and MDA5, respectively. PARP14i treatment reduced IFN-β production approximately threefold during EMCV infection but had no effect on SeV-induced IFN-β production in BMDMs (Fig. 3D) and A549 cells (Fig. 3E). These results indicate that PARP14 preferentially promotes MDA5 but not RIG-I-dependent IFN-β production under these conditions.

### RNAseq analysis identifies over 100 genes up-regulated by PARP14

**Fig 4:**
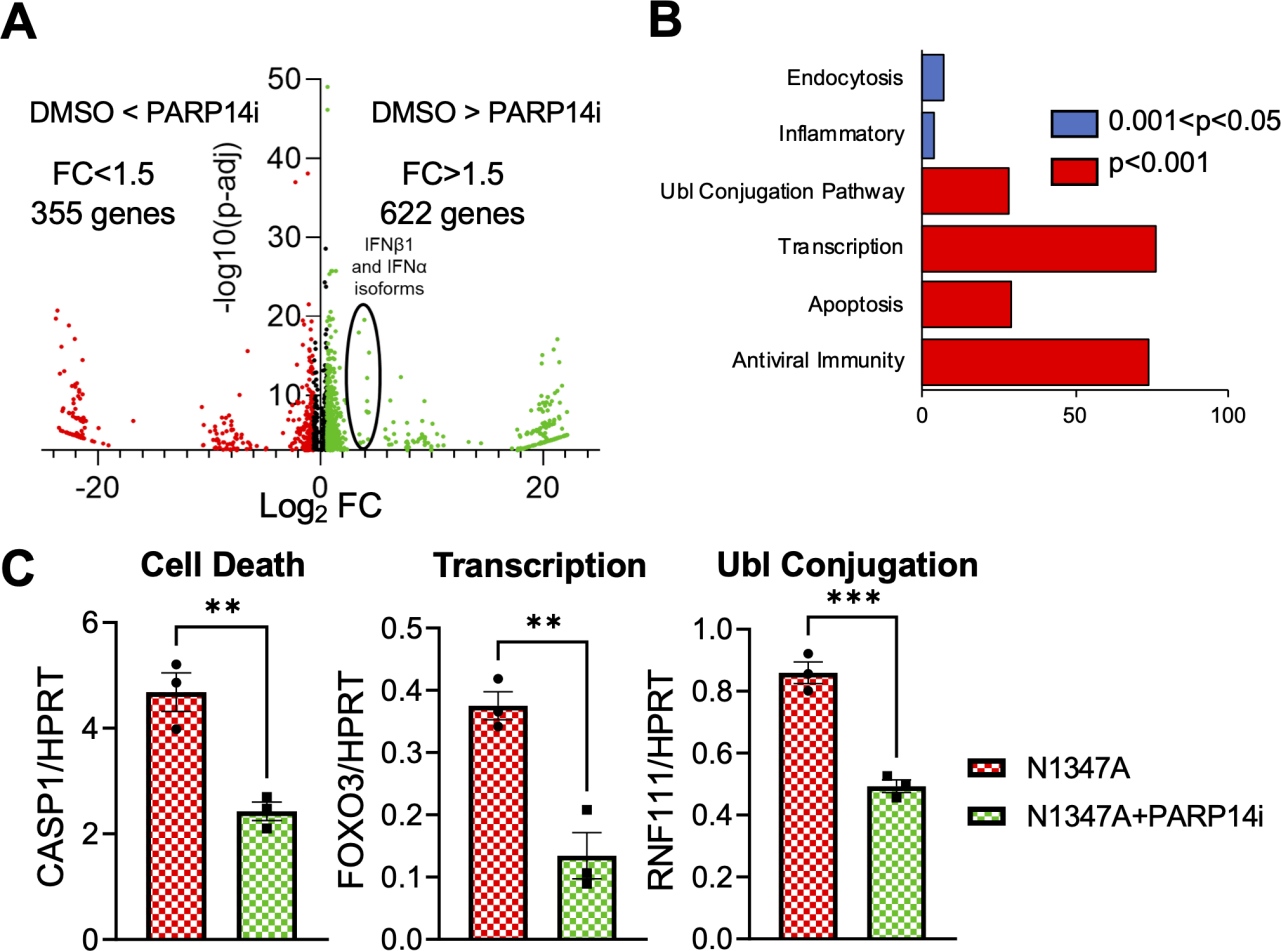
PARP14 regulates the transcription of dozens of genes with multiple biological functions following MHV-N1347A infection of BMDMs. (**A**) WT BMDMs were infected with N1347A MHV at an MOI of 0.1 and then treated with DMSO or 100 nM PARP14i at 1 hpi. RNA was collected from these cells at 12 hpi, and RNAseq was performed to quantify the transcriptome with and without PARP14i. The ratio of gene expression in the presence or absence of PARP14i treatment was plotted against the minus log_10_ value of the *P*-adjusted value as a volcano plot. (**B**) Gene ontology analysis/pathway enrichment analysis of genes that were identified to be promoted by PARP14 catalytic activity was carried out using the DAVID gene ontology tool. Pathways enriched with a *P*-value of less than 0.05 were plotted. (**C**) WT BMDMs were infected with MHV N1347A at an MOI of 0.1 and treated with DMSO or 100 nM PARP14i at 1 hpi. RNA was isolated from cells at 12 hpi, and CASP1, FOXO3, and RNF111 mRNAs were quantified by qPCR using the ΔCt method. RNAseq data shown in (**A, B**) are from one experiment with N = 3. Data shown in (**C**) are from one experiment and are representative of two independent experiments with N = 3 biological replicates per group for each experiment. Data in (**C**) were tested for statistical significance using a standard *t*-test between the indicated samples.

Next, we used RNAseq to identify all genes that are influenced by PARP14-dependent MARylation, in addition to IFNs and ISGs. To identify PARP14-regulated genes, we analyzed the transcriptome of BMDMs that are infected by N1347A with and without PARP14i. As expected from previous studies, N1347A-infected DMSO-treated BMDMs had elevated levels of IFN-I, ISGs, and dozens of other genes involved in antiviral immunity. Upon treatment with PARP14i, the mRNA levels of a substantial number of these genes were reduced, indicating that these mRNAs are increased by PARP14-dependent MARylation. Overall, we found that 622 genes were upregulated by PARP14, while 355 genes were downregulated, for a total number of 977 differentially expressed genes expressed above a threshold fold change (FC) of 1.5-fold and threshold *P*-adjusted value of 0.001 (Fig. 4A). Next, we performed a functional analysis on the genes that are regulated by PARP14 MARylation activity. Many of the genes promoted by PARP14 were genes involved in antiviral immunity (Fig. 4B). However, genes involved in apoptosis, transcription, and ubiquitin ligase conjugation were also upregulated. Representative genes from each of these pathways were validated by qPCR (Fig. 4C). Furthermore, we took a closer look at some of the genes observed to be upregulated or downregulated over 20-fold. These genes were found to cluster in biological processes of transcription and transcriptional regulation with a very low *P*-value (upon functional enrichment analysis by Database for Annotation, Visualization and Integrated Discovery [DAVID]). Overall, this data suggests that PARP14 is an important regulator of antiviral immunity but may also regulate other cellular processes such as cell death and transcription.

### PARP14 is required for the repression of Mac1-mutant coronavirus replication

**Fig 5:**
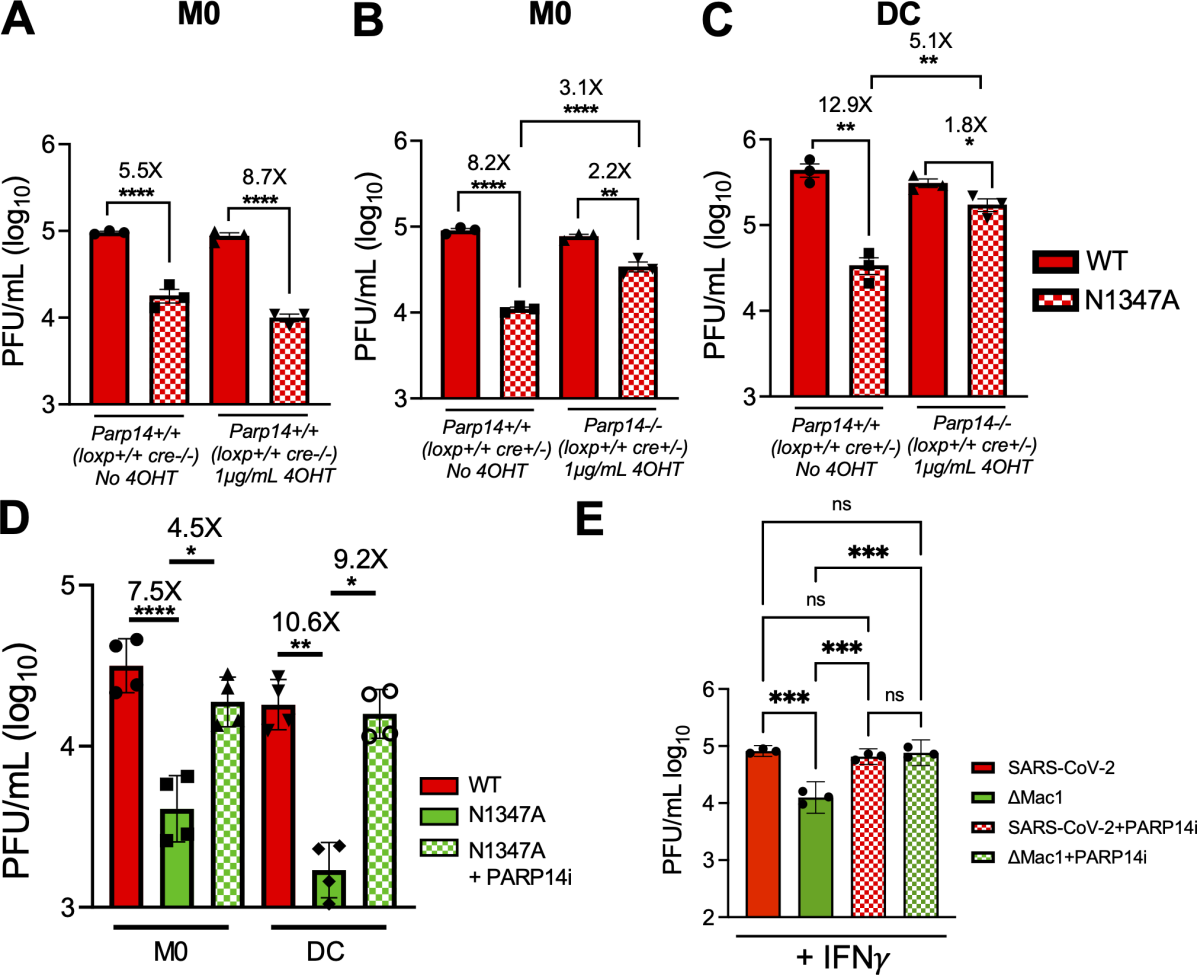
PARP14 inhibits the replication of Mac1-mutant MHV and SARS-CoV-2. (**A**) *Parp14* floxed *cre^+/−^* BMDMs were treated with and without 4-OHT. Twenty-four hours later, these cells were infected with WT and N1347A MHV at an MOI of 0.1. At 20 hpi, cells and supernatants were collected, and progeny virus was quantified by plaque assay. Data shown in (**A**) are from one experiment and are representative of two independent experiments with N = 3 biological replicates per group for each experiment. (**B, C**) PARP14 floxed *cre^+^* BMDMs (**B**) and DCs (**C**) were treated with or without 4-OHT. Twenty-four hours later, these cells were infected with WT and N1347A MHV at an MOI of 0.1. At 20 hpi, cells and supernatants were collected, and progeny virus was quantified by plaque assay. (**D**) PARP14^+/+^ BMDMs and DCs were infected with WT and N1347A MHV at an MOI of 0.1 and then treated with DMSO or PARP14i (1 μM). At 20 hpi, cells and supernatants were collected, and progeny virus was quantified by plaque assay. (**E**) WT A549-ACE2 cells were treated with IFN-γ (50 units) O/N and then infected with WT (red bars) and ΔMac1 (green bars) SARS-CoV-2 at an MOI of 0.1 and treated with DMSO or PARP14i (1 μM) at 1 hpi. At 48 hpi, cells and supernatants were collected and progeny virus was quantified by plaque assay. Data shown in (**B–E**) are from one experiment and are representative of three independent experiments with N = 3 or 4 biological replicates per group for each experiment. Data in (**A–E**) were tested for statistical significance using a one-way ANOVA.

We previously found that *parp14* knockdown mildly increased the replication of MHV-N1347A in BMDMs; however, congenital *parp14* KO mice did not have increased MHV-N1347A replication (3). To determine if PARP14 was required to repress MHV-N1347A, we infected our *parp14^−/−^* and *parp14^+/+^* BMDMs as described above. As previously observed, MHV-N1347A had a significant growth defect compared to MHV-WT virus in *parp14^+/+^* cells, regardless of whether the cells were or were not treated with tamoxifen (Fig. 5A). Interestingly, *parp14^−/−^* mostly restored the replication of MHV-N1347A, suggesting that PARP14 is required for restricting MHV-N1347A virus replication in BMDMs (Fig. 5B). We also found that MHV-N1347A replication was increased to levels similar to those of MHV-WT in *parp14^−/−^* DCs (Fig. 5C). To test whether this increased MHV-N1347A replication in *parp14^−/−^* cells is due to PARP14-dependent MARylation, we measured MHV-N1347A replication in the presence and absence of PARP14i. In the presence of PARP14i, MHV-N1347A replication was similarly restored to near MHV-WT levels but did not have a significant effect on the replication of MHV-WT, suggesting that the catalytic activity of PARP14 was required for restricting MHV-N1347A replication (Fig. 5D; Fig. S4A and B).

We next tested whether PARP14 was required to restrict the replication of SARS-CoV-2 ΔMac1. We previously found that SARS-CoV-2 ΔMac1 replicates near SARS-CoV-2 levels in Calu-3 cells, but that SARS-CoV-2 ΔMac1 is more sensitive to IFN-γ pre-treatment than WT virus, and IFN-γ induces the expression of several PARPs, including PARP14 (4). To determine if PARP14 is required for IFN-γ-mediated restriction of SARS-CoV-2ΔMac1, we infected IFN-γ pretreated A549-ACE2 cells with SARS-CoV-2 WT and ΔMac1 with and without PARP14i. Upon treatment with PARP14i, we observed that the WT virus was not impacted either in the presence (Fig. 5E) or absence (Fig. S4C) of IFN-γ; in contrast, the replication of SARS-CoV-2 ΔMac1 in cells pre-treated with IFN-γ was significantly increased to levels near that of SARS-CoV-2 WT virus (Fig. 5E). Overall, these results suggest that MARylation triggered by PARP14 is required to restrict the replication of Mac1-deficient MHV and SARS-CoV-2 viruses.

### PARP14 restricts HSV-1 replication

**Fig 6:**
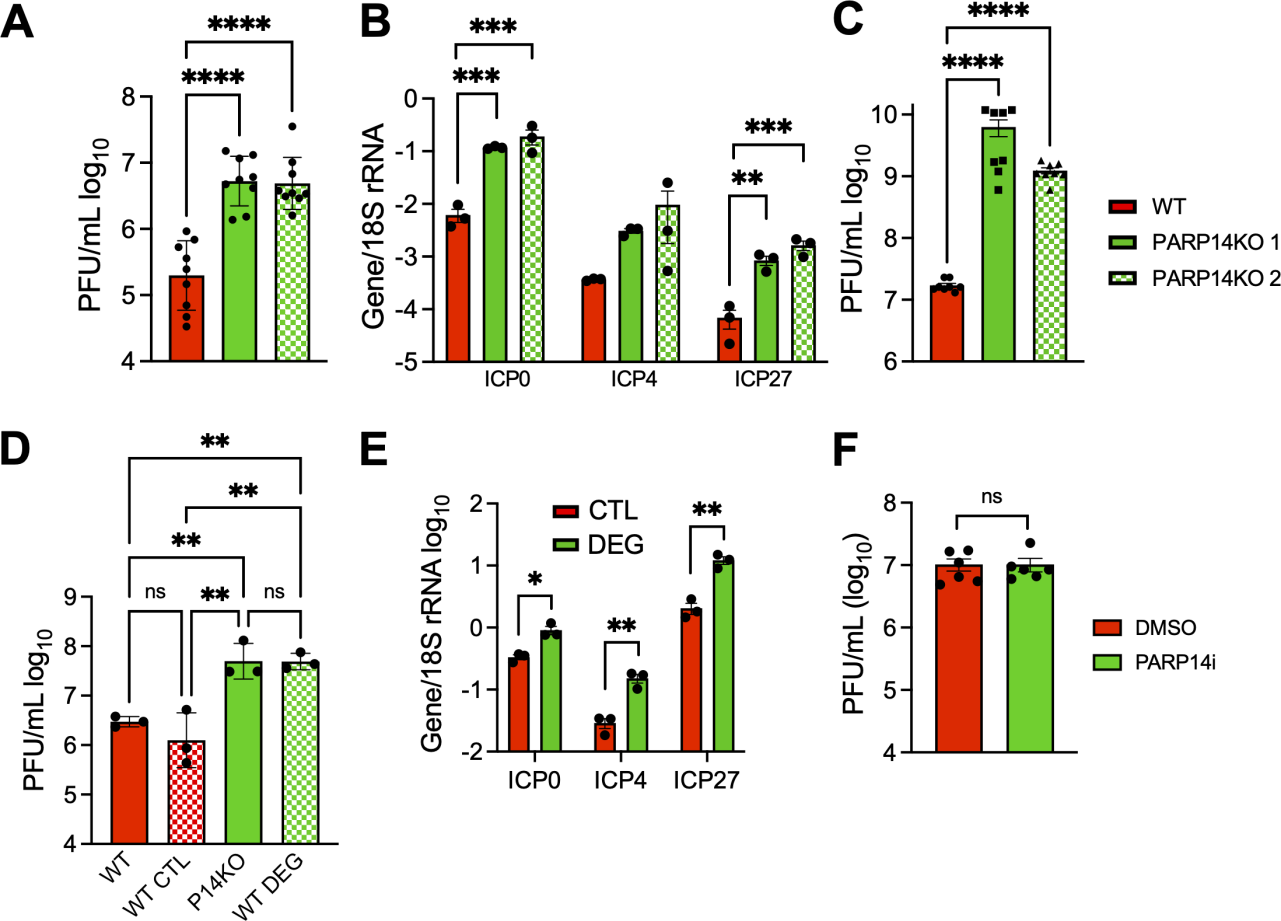
PARP14 restricts the replication of HSV-1 in A549 cells. (**A**) WT and PARP14 KO A549 cells were infected with HSV-1 at an MOI of 0.1. Cells and supernatants were collected 24 hpi, and progeny virus was quantified by plaque assay on Vero cells. The results in (**A**) are the combined results of three independent experiments, N = 9 biological replicates per group. (**B**) WT and PARP14 KO A549 cells were infected with HSV-1 at an MOI of 0.1. At 24 hpi, RNA was harvested from infected cells, and HSV-1 immediate early gene ICP4, ICP0, and ICP27 mRNAs were quantified by qPCR using the ΔCt method, having 18S rRNA abundance as the endogenous control. The data in B is from one experiment representative of two independent experiments, N = 3 biological replicates per group for each experiment. (**C**) WT and PARP14 KO A549 cells were infected with serial dilutions of HSV-1 starting at a concentration of 1.85 × 10^9^ PFU/mL and overlaid with methylcellulose. At 24 hpi, the plaquing efficiency was determined by counting the number of plaques at each dilution. The data in C is the combined results of three separate experiments, N = 9 biological replicates per group. (**D**) WT A549 cells were treated with 1 µM PARP14 degron control (CTL) or PARP14 degron (DEG) overnight. The cells were then infected with HSV-1 at an MOI of 0.1 PFU/cell. At 24 hpi, cells and supernatants were collected at indicated time points, and progeny virus was quantified by plaque assay. The data in (**D**) is from one experiment representative of two independent experiments, N = 3 biological replicates per group for each experiment. (**E**) WT A549 cells were treated and infected as described in (**D**), at 24 hpi, RNA was harvested from infected cells, and HSV-1 immediate early gene ICP4, ICP0, and ICP27 mRNAs were quantified by qPCR using the ΔCt method, having 18S rRNA abundance as the endogenous control. The data in (**E**) is from one experiment representative of two independent experiments, N = 3 biological replicates per group for each experiment. (**F**) WT A549 cells were infected with HSV-1 at an MOI of 0.1 PFU/cell and then treated with DMSO or PARP14i (1 μM) at 1 hpi. Cells and supernatants were collected at 24 hpi, and progeny virus was quantified by plaque assay. The data in (**F**) are the combined results of two independent experiments, N = 6 biological replicates per group. Data in (**A–D**) were tested for statistical significance using a one-way ANOVA, and data in (**E, F**) were tested for statistical significance using a standard *t*-test.

PARP14 is one of several PARPs that have evolved under positive selection in primates, indicating that it is involved in host-pathogen conflict (22). Thus, we hypothesized that in addition to CoVs, it might repress the replication of other viral families. First, we tested the effect of PARP14 deletion on HSV-1, which has been co-evolving with humans for several million years. Furthermore, PARP14 was identified as a host factor that binds to HSV-1 DNA (25, 26). We infected A549 WT and two different clones of PARP14 KO cells (3) with HSV-1 (strain KOS) at an MOI of 0.1 (Fig. 6A) or 0.01 (Fig. S5) and found that replication of HSV-1 was enhanced by over 1–2 logs following infection in PARP14 KO cells, strongly indicating that PARP14 represses HSV-1 replication. HSV-1 infection is characterized by activation of some of its immediate early genes, like ICP0, ICP4, and ICP27. As a separate measure of replication, we infected WT and KO cells with 0.1 MOI HSV-1 and quantified the expression of ICP0, ICP4, and ICP27 mRNA transcripts at 24 hpi. We observed increased gene expression in PARP14 KO cells compared to WT cells, again indicating that PARP14 restricts HSV-1 infection (Fig. 6B). Next, we tested the ability of HSV-1 to initiate a productive infection and spread on A549 WT and PARP14 KO cells using a plaquing efficiency assay. The ability of a virus to produce a plaque indicates its ability to enter cells and initiate a productive infection, which is unique from measuring its ability to replicate. Similar to previous observations, we found that PARP14 KO also enhanced the plaquing efficiency of HSV-1 compared to WT cells by >2 logs on average (Fig. 6C). We then corroborated this observation by quantifying viral yields and viral mRNA levels in A549 cells infected with HSV-1 and treated with the PARP14 degron (DEG) or its corresponding negative control (CTL). Again, we saw an increase in both infectious virus and the ICP0, ICP4, and ICP27 transcripts in DEG-treated cells, confirming that the observed increase in virus replication was due to the absence of PARP14 (Fig. 6D and E). Since the MARylating activity of PARP14 was responsible for restricting CoV replication, we tested the ability of PARP14i to enhance HSV-1 infection. Interestingly, HSV-1 replication was not affected in PARP14i-treated A549 cells compared to untreated cells, suggesting that PARP14 restricted HSV-1 replication in a MARylation-independent manner (Fig. 6F). Overall, these data show that PARP14 restricts HSV-1 infection and does so in an ADP-ribosylation-independent manner.

### PARP14 promotes the replication of vesicular stomatitis virus

**Fig 7:**
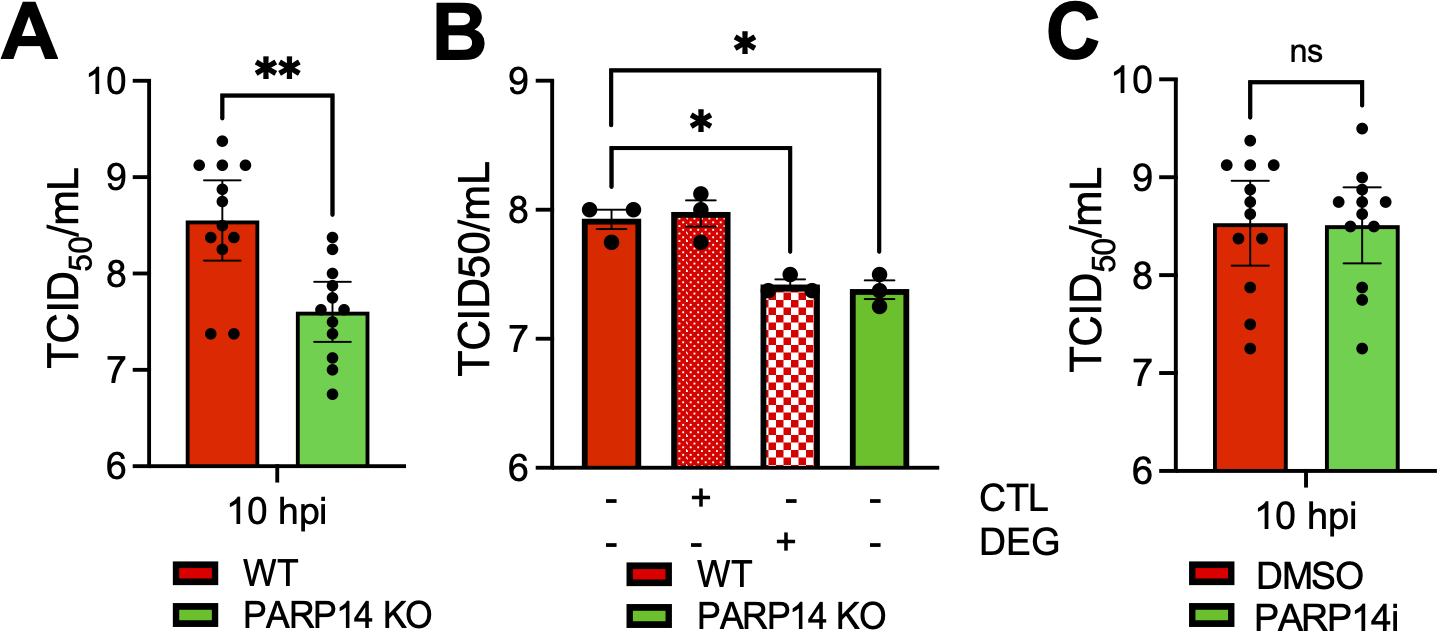
PARP14 is required for efficient replication of VSV-GFP in cell culture. (**A**) A549 WT and PARP14 KO cells were infected with VSV-GFP at an MOI of 1. At 10 hpi, cells and supernatants were collected, and progeny virus was quantified by 50% tissue culture infectious dose (TCID_50_). The results in A are the combined results of four independent experiments, N = 12 biological replicates per group. (**B**) A549-ACE2 WT and PARP14 KO cells were infected with VSV-GFP at 1 MOI and then treated with 1 µM PARP14 degron control (CTL) or PARP14 degron (DEG) at 1 hpi. At 10 hpi, cells and supernatants were collected, and progeny virus was quantified by TCID_50_. The results in (**B**) are from one experiment representative of two independent experiments, N = 3 biological replicates per group for each experiment. (**C**) A549 WT cells were infected with VSV at an MOI of 1 and then treated with DMSO or 100 nM PARP14i (P14i) at 1 hpi. At 10 hpi, cells and supernatants were collected, and progeny virus was quantified by TCID_50_. The results in (**C**) are the combined results of four independent experiments, N = 12 biological replicates per group. Data in (**A**) and (**C**) were tested for statistical significance using a non-parametric *t*-test, while data in (**B**) were tested for statistical significance using a one-way ANOVA.

Next, we tested the ability of PARP14 to restrict the replication of additional types of RNA viruses. First, we tested the infectivity of lymphocytic choriomeningitis virus clone-13 (LCMV-cl13), an ambisense RNA virus, in WT and PARP14 KO BMDMs. To measure the infectivity of LCMV-cl13 in BMDMs, we measured the amount of LCMV nucleoprotein (LCMV-NP) using a fluorescently labeled antibody against LCMV-NP (clone anti-VL4) and intracellular flow cytometry. PARP14 KO cells had comparable levels of LCMV-NP+ cells compared to WT cells, ~4%–5%. These data indicate that LCMV-cl13 infection is not impacted by PARP14 (Fig. S6A and B). To further explore the role of PARP14 in the replication of negative-sense RNA viruses, we tested the ability of VSV-GFP to replicate in PARP14 KO A549 cells. We infected A549 WT and PARP14 KO cells with VSV-GFP at an MOI of 1 and measured the infectious virus produced at 10 hpi. Interestingly, VSV-GFP replication was significantly reduced in A549 PARP14 KO cells compared to WT cells, indicating that PARP14 promotes VSV-GFP replication (Fig. 7A). To confirm this result, we created a new set of A549 WT and PARP14 KO cells using separate control and PARP14 targeting guide RNAs (Fig. S7). Again, we found that VSV replication was significantly reduced in these cells (Fig. 7B). Furthermore, the addition of the PARP14 degrader (DEG) significantly reduced the replication of VSV in WT cells, while the CTL compound had no effect (Fig. 7B). To determine if these differences could be due to an increase in IFN levels, we measured the level of IFN following infection with VSV in both PARP14 KO and PARP14i treated cells. We consistently found no substantial differences in IFN production following VSV infection in PARP14-depleted cells (Fig. S8A). To further confirm that there was no impact of IFN on virus replication, we treated A549 cells with a JAK inhibitor (JAKi) that has previously been used to ablate the production of ISGs (27, 28). While the JAKi reduced the levels of PARP14, an ISG (Fig. S8B), it did not lead to increased replication of VSV in WT or PARP14 KO cells (Fig. S8C). Finally, we tested whether the ADP-ribosyltransferase activity of PARP14 supported VSV replication by treating A549 WT cells with PARP14i. PARP14i treatment did not reduce VSV replication, indicating that the proviral function of PARP14 was independent of its MARylating activity (Fig. 7C). Overall, these results suggest that PARP14 can inhibit the replication of CoVs and HSV-1 but enhance the replication of VSV, a negative-sense RNA virus.

## DISCUSSION

ADP-ribosylation has rapidly gained attention recently due to its involvement in several key cellular processes. It is also implicated in the replication and pathogenesis of several viruses and the innate immune response. PARP1 and PARP5-catalyzed poly ADP-ribosylation (PARylation) was shown to restrict the replication of DNA viruses such as Epstein-Barr virus and Kaposi’s sarcoma virus by PARylating key replication and latency proteins (11, 29). More recently, PARP12-mediated MARylation of Zika virus proteins such as NS1 and NS3 leads to its proteolytic degradation, restricting Zika virus replication and its subsequent infection (30). The ability of Chikungunya virus and CoV to bind and reverse ADP-ribosylation was also shown to be critical for the replication of those respective viruses, highlighting the potential antiviral role of ADP-ribosylation during CHIKV and CoV infection (4, 31, 32). Furthermore, PARP10 was shown to inhibit the production of alphavirus nsp3 by targeting polyprotein processing (33). Apart from directly affecting virus replication, PARPs also play an important role in modulating host antiviral processes such as IFN production. PARP9 promotes STAT1 signaling (16), while other PARPs such as PARP7, PARP11, PARP5, and PARP10 also restrict IFN and NF-κB responses by targeting proteins such as TBK1, IFNAR, MAVS, and NEMO, respectively (7, 8, 34, 35). It is now abundantly clear that PARP-mediated ADP-ribosylation is pivotal for innate immune responses, and hence it is essential to understand how each PARP impacts the antiviral response.

PARP14 has emerged as a critical host factor that impacts multiple immune signaling pathways, though its role in virus infection remains largely unknown. It was initially found to bind STAT6 and promote IL4-dependent anti-inflammatory gene regulation (15). This function of PARP14 promotes T_H_2 gene expression and IgE production in immune cells (36). Recently, a PARP14 inhibitor (RBN-3143) was reported to mitigate the effects of allergen-induced IgE production and overproduction of mucus, reduce T_H_2 cell abundance, and restore αPD-1 sensitivity to tumors (37). PARP14 also inhibits STAT-1 phosphorylation by MARylating key residues, and knockdown or knockout of PARP14 increased pro-inflammatory cytokines in primary human and mouse M1, but not M2 macrophages (16). In contrast, PARP14 promoted IFN-I and pro-inflammatory cytokine production in RAW 264.7 cells following LPS treatment and *S. typhimurium* infection (17). PARP14 also promoted IFN-I production in response to MHV infection and poly(I:C) stimulation in BMDMs and A549 cells, respectively (3). In this study, we demonstrated that the enzymatic activity of PARP14 promotes MDA5-dependent IFN-I and IFN-III production when stimulated with a dsRNA analog poly(I:C), EMCV, or CoV infection by directly quantifying IFN-I and IFN-III mRNA and protein levels in multiple cell lines. In general, the impact of PARP14 on innate and adaptive immune pathways is cell type and condition-specific. It remains unclear if PARP14 both enhances IFN-I and IFN-III production and limits IFN signaling in the same cells, or if it only represses IFN signaling in M1 macrophages. It is feasible that PARP14 may dampen IFN signaling to control its own ability to stimulate IFN production. The mechanism of how PARP14 regulates MDA-5-dependent IFN production remains unclear and is currently being investigated.

Interestingly, RIG-I had little to no impact on IFNβ production in BMDMs following infection with MHV (Fig. 3A). These results are consistent with others that have demonstrated that MDA5 is largely responsible for IFN production in brain macrophages and microglia in response to MHV, as well as in Calu-3 and human lung fibroblasts in response to SARS-CoV-2 (38–41). MDA-5 is also critical for protection from severe COVID-19, as individuals with MDA-5 mutations are more likely to get severe COVID (42, 43). However, this does not mean that RIG-I is unable to induce IFN during CoV infection. RIG-I can induce IFN in response to MHV in oligodendrocytes and is largely responsible for SARS-CoV-2-induced IFN induction in intestinal epithelial cells (44, 45). Furthermore, the CoV N protein can block RIG-I-dependent responses, indicating that RIG-I-induced IFN induction may be masked during CoV infection (46–48). Thus, our results have further confirmed that MDA-5 is a major sensor for CoVs in macrophages, but don’t discount the likelihood that RIG-I contributes to IFN induction following CoV infection in certain contexts.

In addition to inducing IFN, PARP14 expression is stimulated by IFN and virus infection, and its ADP-ribosyltransferase activity triggers the production of IFN-induced ADP-ribose bodies (ICABs) (3). ICABs are regions of the cytoplasm with a punctate ADP-ribose signal that have been observed by immunofluorescence following IFN-γ or poly(I:C) treatment (18–21, 49). In addition, increased mono-ADP-ribosylation is visible by western blotting following the same treatments in A549 cells (18). Importantly, both signals were dependent on the catalytic activity of PARP14 and can be reversed by macrodomain proteins, including the SARS-CoV-2 Mac1 protein (18, 49). These bodies also contain PARP9, DTX3L, and p62 and also overlap with ubiquitin staining. Furthermore, DTX3L and p62 are ADP-ribosylated by PARP14, likely within ICABs, and are required for the maintenance of the ICABs (18, 20, 21, 50). Despite this, there is no current data showing that PARP14 or other components of ICABs restrict virus infection. Here, we found that the catalytic activity of PARP14 was required to repress the replication of Mac1-mutant CoVs (Fig. 5). Furthermore, our findings that PARP14 catalytic activity is required for the repression of SARS-CoV ΔMac1 only in the presence of IFN-γ strongly suggest that ICABs play a key role in the repression of CoV replication. Future studies will determine if p62, DTX3L, or other PARP14 targets within ICABs restrict CoV replication and identify the mechanisms by which they do so.

PARP14 is one of the four PARPs that have evolved under positive selection, further indicating that it is likely involved in host-pathogen conflict (22). In addition to restricting Mac1-mutant CoVs, we also found that it was required for repression of HSV-1 (Fig. 6). HSV-1 and other herpesviruses have been evolving with mammals for millions of years, so it is more likely that PARP14 is co-evolving with herpesviruses than CoVs. Finally, we found that PARP14 enhances the replication of VSV in an ADP-ribosylation-independent manner. PARP14 interacts with SARS-CoV-2 RNA (51) and HSV-1 DNA (25); thus, the mechanism by which PARP14 restricts and promotes virus replication could be through interactions with nucleic acids. In fact, PARP14 bound to the HSV-1 genome, in association with other DNA repair proteins, as early as 3 hours post-infection, suggesting that PARP14 possibly binds to DNA to inhibit transcription and/or DNA replication (25, 26). These interactions may help explain the positive selection of PARP14, as it may need to rapidly evolve to mutations in DNA or RNA sequences within viral genomes. However, it is unclear if PARP14 interacts with HSV-1 DNA directly or through its interactions with other proteins.

PARP14 is a large protein (203 kDa) with multiple domains (12). The ADP-ribosyl transferase (ART) domain of PARP14 binds to NAD^+^ and transfers an ADP-ribose moiety to its targets. Its ART domain has an HYL (hydrophobic residue-tyrosine-leucine) domain as part of the catalytic triad, which restricts its ART activity to MARylation (52, 53). PARP14 also has three tandem macrodomains (MD), of which MD1 has ARH activity against MARylated protein and nucleic acid substrates, making it a rare enzyme that both adds and reverses the same post-translational modification (13, 14). PARP14 also has a WWE domain that can bind to ADP-ribose subunits (54), several KH domains that facilitate protein and nucleic acid binding, and several RNA recognition motifs (RRMs) (12). However, the roles and substrates of these domains, especially during virus infection, remain largely unknown. Given the potential of PARP14 to bind and modify protein and nucleic acid substrates through its many domains, identifying the protein and/or nucleic acid binding partners of PARP14 during infection and the domains required for these interactions could reveal several novel mechanisms by which PARP14 regulates virus replication and pathogenesis. 

## MATERIALS AND METHODS

### Biosafety statements

All work with SARS-CoV-2 was conducted in the University of Kansas EHS-approved BSL-3 facility following standard operating procedures. Animal studies were approved by the Institutional Animal Care and Use Committees at the University of Iowa and the University of Kansas and met stipulations of the Guide for the Care and Use of Laboratory Animals.

### Cell culture and reagents

NHDF, 293T, HeLa-MVR, A549, A549-PARP14KO, A549-ACE2 (a generous gift from Susan Weiss, University of Pennsylvania), 17Cl-1, and Vero E6 cells were grown in Dulbecco’s modified Eagle medium (DMEM) supplemented with 10% fetal bovine serum (FBS). Calu-3 cells (ATCC) were grown in MEM supplemented with 20% FBS. Baby hamster kidney cell line BSR-T7/5, constitutively expressing T7 RNA polymerase, was maintained in Glasgow’s Minimum Essential Medium (G-MEM) with 10% FBS. Bone marrow-derived cells were grown in RPMI supplemented with 10% FBS and differentiated into M0 macrophages using M-CSF (Millipore-Sigma) and dendritic cells (DCs) using GM-CSF (Millipore-Sigma). All cell lines were grown at 37°C and 5% CO_2_. IFN-γ was purchased from R&D Systems, and poly(I:C) HMW (0.5 µg/mL) was purchased from Millipore-Sigma. RBN012759 (PARP14i) was purchased from Atomwise. RBN012811 (DEG) and its associated negative control RBN013527 (CTL) were generously provided by Dr. Mario Niepel (Ribon Therapeutics). Cells were transfected with either Polyjet (Amgen) or Lipofectamine 3000 (Fisher Scientific) per the manufacturer’s instructions.

### Creation of inducible PARP14 knock-out mice

#### Animals

The insertion of loxP sites flanking exon 2 of PARP14 was carried out by the University of Iowa genome editing facility. C57BL/6J mice were purchased from Jackson Labs (000664; Bar Harbor, ME). Male mice older than 8 weeks were used to breed with 3–5-week-old super-ovulated females to produce zygotes for pronuclear injection. Female ICR (Envigo; Hsc:ICR[CD-1]) mice were used as recipients for embryo transfer. All animals were maintained in a climate-controlled environment at 25°C and a 12/12 light/dark cycle.

#### Preparation of Cas9 RNPs and the injection mix

Chemically modified CRISPR-Cas9 crRNAs targeting regions 5′ and 3′ of exon 2 of PARP14 (Table S1) and CRISPR-Cas9 tracrRNA were purchased from IDT (Alt-R CRISPR-Cas9 crRNA; Alt-R CRISPR-Cas9 tracrRNA [Cat# 1072532]). The crRNAs and tracrRNA were suspended in T10E0.1 and combined to a 1 ug/uL (~29.5 uM) final concentration in a 1:2 (μg:μg) ratio. The RNAs were heated at 98°C for 2 minutes and allowed to cool slowly to 20°C in a thermal cycler. The annealed cr:tracrRNAs were aliquoted to single-use tubes and stored at −80°C. Cas9 nuclease was also purchased from IDT (Alt-R S.p. HiFi Cas9 Nuclease). Cr:tracr:Cas9 ribonucleoprotein complexes were made by combining Cas9 protein and cr:tracrRNA in T10E0.1 (final concentrations: 250 ng/μL (~1.6 μM) Cas9 protein and 200 ng/μL (~6.1 μM) cr:tracrRNA). The Cas9 protein and annealed RNAs were incubated at 37°C for 10 minutes. The RNP complexes were combined with a single-stranded repair template, containing the loxP sites to be inserted around exon 2, and incubated for an additional 5 minutes at 37°C (single-stranded repair template sequence available upon request). The concentrations in the injection mix were 50 ng/μL (~0.3 μM) Cas9 protein and 20 ng/μL (~0.6 μM) each cr:tracrRNA and 40 ng/μL single-stranded repair template.

#### Collection of embryos and injection

Pronuclear-stage embryos were collected using methods described in reference 55. Embryos were collected in KSOM media (Millipore; MR101D) and washed three times to remove cumulus cells. Cas9 RNPs and double-stranded repair template were injected into the pronuclei of the collected zygotes and incubated in KSOM with amino acids at 37°C under 5% CO_2_ until all zygotes were injected. The CRISPR injection protocol was previously described (56). Fifteen to 25 embryos were immediately implanted into the oviducts of pseudo-pregnant ICR females. Offspring were heterozygous for the loxP insertions. These mice were sequentially bred to result in floxed homozygote mice. Genotyping was performed using PCR amplification with primers listed in Table S2 and restriction digestion by EcoR1. These were then crossed with C57BL/6.Cg-Ndor1^Tg(UBC-cre/ERT2)1Ejb^/IJ mice (Jax strain # 007001) till mice colonies that were homozygous for *loxP* sites and heterozygous for the *cre* gene were confirmed by genotyping. The resulting mice were backcrossed twice before using these mice for the experiments described in this study.

### Creation of PARP14 knock-out A549-ACE2 cells

A549-ACE2 cells were cultured in DMEM supplemented with 10% FBS at 37°C with 5% CO₂. For CRISPR/Cas9 transfection, cells were seeded in 6-well plates at 2 × 10⁵ cells per well before transfection. At 40%–80% confluency, cells were transfected with either PARP14 CRISPR/Cas9 KO plasmid (sc-402812, Santa Cruz Biotechnology) or control CRISPR/Cas9 plasmid (sc-418922, Santa Cruz Biotechnology) using PolyJet transfection reagent according to the manufacturer’s instructions. After 72 hours of incubation, successful transfection was confirmed by GFP expression via fluorescence microscopy. GFP-positive cells were isolated using a BD FACSymphony cell sorter, and single cells were sorted into individual wells to establish monoclonal populations. WT control and PARP14 knockout expanded clones were validated by western blot analysis using anti-PARP14 mouse monoclonal antibody (sc-377150, Santa Cruz Biotechnology).

### Virus infections

#### MHV-JHM^IA^

The recombinant WT and N1347A MHV JHM^IA^ virus used in this study has been previously reported (3, 57, 58). Virus stocks were grown in 17Cl-1 cells and titered by plaque assay in HeLa-MVR cells. BMDMs and DCs were infected at an MOI of 0.1 PFU/cell for the indicated periods of time after a 60 minute adsorption phase. Virus titers were determined by plaque assay on HeLa-MVR cells.

#### SARS-CoV-2

Recombinant SARS-CoV-2 WT and ΔMac1 virus based on the original Wuhan isolate used in this study has been previously reported (4). Virus stocks were grown and titered by plaque assay in VeroE6 cells. A549-ACE2 cells were infected at an MOI of 0.1 for 48 hours after a 60 minute adsorption phase. Calu3 cells were infected at an MOI of 0.1 for 48 hours after a 120 minute adsorption phase and Trypsin-TCPK treatment (1 µg/mL). Virus titers were determined in Vero E6 cells using a plaque assay. All the SARS-CoV-2 experiments were carried out under BSL-3 conditions at the University of Kansas, following approved SOPs.

#### HSV-1

Stocks of the HSV-1 strain KOS were propagated and titered as previously described (59). For viral yield assays, A549 and A549-PARP14KO cells (5 × 10^4^) were seeded in triplicate in a 24-well plate and infected at an MOI of 0.1 PFU/cell with HSV-1. At 1 hpi, cells were washed twice with PBS to remove unabsorbed virus. Infected cells were harvested and collected 24 hpi and titered by standard plaque assays on Vero cells. For PARP14 inhibitor experiments, A549 cells were treated with or without the PARP14 inhibitor, and infections were carried out as described for the HSV-1 yield assays. To determine plaquing efficiencies, A549 and A549-PARP14KO cells were plated in triplicate in 12-well plates and infected with serial dilutions from HSV-1 stocks. The wells were then covered in methylcellulose, and after 3 days, the cells were fixed and stained. The relative titers on each cell line were determined by counting plaques in each well and multiplying by the dilution factor. To assess virus transcript levels, A549 cell lines (5 × 10^4^) were plated in triplicate in a 24-well plate. Cells were infected at an MOI of 0.1 with HSV-1 and washed twice with PBS 1 hpi. At 6 hpi, media were removed, and cells were resuspended in TRIzol reagent (250 µL). RNA was isolated according to the manufacturer’s protocol and reverse transcribed as published (ref). cDNAs of viral and cellular transcripts were amplified using primers for ICP0, ICP4, ICP27, and 18S rRNA as previously described (60).

#### VSV

VSV-GFP Indiana strain was generously provided by Dr. Asit Pattnaik (University of Nebraska-Lincoln) (61). Virus stocks were grown and titered by plaque assay in VeroE6 cells. A549 cells were infected with VSV-GFP at an MOI of 1 PFU/cell. Infected cells were then incubated and collected at 10 hpi after a 60 minute adsorption phase. Virus titer was determined by 50% tissue culture infectious dose (TCID_50_) assay in Vero E6 cells.

#### LCMV

LCMV Clone-13 (LCMV-cl13) virus stocks were grown in BHK cells and titered by plaque assay in Vero E6 cells. BMDM cells were infected with LCMV-cl13 at an MOI of 1–6. Infected cells were then incubated and collected at 24 hpi. For surface staining, samples were treated with Fc block (CD16/32, eBioscience) and then incubated with Tonbo Ghost Viability dye (violet 510, Tonbo Biosciences), F4/80 (Pacific orange, Invitrogen, CLONE), and LCMV-NP (clone VL4 [BioXcell], self-conjugated to Alexa Fluor 488 following the manufacturer’s instructions, [Thermo Fisher Scientific]). All flow cytometry data were analyzed using FlowJo software (BD Biosciences).

### Immunoblotting

Total cell extracts were lysed in sample buffer containing SDS, protease and phosphatase inhibitors (Roche), β-mercaptoethanol, and a universal nuclease (Fisher Scientific) and boiled at 95°C for 5 minutes. Proteins were resolved on an SDS polyacrylamide gel, transferred to a polyvinylidene difluoride membrane, hybridized with a primary antibody, reacted with an infrared (IR) dye-conjugated secondary antibody, visualized using a Li-COR Odyssey Imager (Li-COR), and analyzed using Image Studio software. Primary antibodies used for immunoblotting included anti-SARS-CoV-2 N (SinoBiological 40143-R001, 1:5,000), anti-OAS3 (Cell Signaling 41440, 1:250), anti-PARP14 (Santa Cruz Biotechnology SC-377150, 1:100), and anti-GAPDH (Millipore-Sigma G8795, 1:5,000) antibodies. Secondary IR antibodies were purchased from Li-COR.

### PARP14 cellular ADP-ribosylation assays

293T cells were transfected with GFP-PARP14 WT or GFP-PARP14 G832E plasmid using the jetOPTIMUS transfection reagent with 0.4 µg DNA/well and with a ratio of 1:1 µL transfection reagent: µg DNA. The media was exchanged 4 hours post-transfection. The next day, cells were dosed with RBN012759 for 4 hours. Cells were washed once with cold PBS, and the plate was frozen at −80°C prior to lysis. Samples were thawed on ice, lysed with a cytosolic lysis buffer (50 mM HEPES pH 7.4, 150 mM NaCl, 1 mM MgCl_2_, 1% Triton X-100 supplemented with fresh 1 mM TCEP, 1× cOmplete EDTA-free Protease Inhibitor Cocktail [Roche] and 30 µM Phthal01 [panPARP inhibitor] [62]), and clarified by centrifugation at 14,000 rpm at 4°C for 10 minutes. The supernatant containing total protein was quantified using the Bradford assay reagent (Bio-Rad). Lysates were normalized, and sample buffer was added to 1× (10% glycerol, 50 mM Tris-Cl [pH 6.8], 2% SDS, 1% β-mercaptoethanol, 0.02% bromophenol blue). Samples were boiled at 95°C for 5 minutes and resolved in 4%–20% Mini-PROTEAN TGX Precast Protein Gels (Bio-Rad). Proteins were transferred to a nitrocellulose membrane and probed overnight at 4°C with primary antibodies, followed by incubation with goat anti-rabbit (1:10,000, Jackson Immuno Research Labs) or goat anti-mouse (1:5,000, Invitrogen) HRP-conjugated secondary antibodies. The blots were then incubated with SuperSignal West Pico substrate (Thermo Scientific) and imaged on a ChemiDoc Gel Imaging System (Bio-Rad). Primary antibodies used for this experiment were Poly/Mono-ADP ribose antibody (Cell Signaling Technology, E6F6A, 1:2,000), Tubulin (Cell Signaling Technology, DM1A, 1:2,000), GFP (Proteintech, pabg1, 1:1,000), Mono-ADP-Ribose antibody 33204 (Bio-Rad, HCA354, 2 µg/mL), and PARP14 (Millipore Sigma, HPA012063, 1:1,000).

### Real-time quantitative polymerase chain reaction analysis

RNA was isolated from respective cells using Trizol (Invitrogen). Briefly, cells were collected at the indicated time points in Trizol (Invitrogen), and RNA was isolated as per the manufacturer’s instructions. cDNA was prepared using M-MuLV reverse transcriptase per the manufacturer’s instructions (NEB). qPCR was performed using PowerUp SYBR green master mix (Applied Biosystems) and primers listed in Table S3. Cycle threshold values were normalized to hypoxanthine phosphoribosyltransferase, glyceraldehyde-3-phosphate dehydrogenase (GAPDH), or 18S rRNA levels using the ΔCt method.

### RNAseq

RNA was isolated from infected BMDMs with and without treatment, isolated from C57BL6 mice as described above. Library preparation with indexing was performed by the University of Kansas Genome Sequencing core facility with the NEB Next RNA Library kit (NEB). RNAseq was performed using an Illumina NextSeq2000 high-output system with paired-end reads of 50 bp. DESeq2 was used to identify DEGs between the BMDM without treatment and MHV-JHM N1347A infected and BMDM with 100 nM PARP14 inhibitor treatment and N1347A infected samples, using simply “treatment” as a factor. DEGs were identified based on the false-discovery rate corrected *P*-value (*P*_ADJ_) and log_2_-fold-change of (log_2_FC) between the samples. Genes were considered up-regulated in an MHV-JHM-infected sample if *P*_ADJ_ <0.05 and log_2_FC >0.6, which is nearly equivalent to a 1.5-fold increase. Similarly, genes were considered down-regulated if *P*_ADJ_ <0.05 and log_2_FC <−0.6, or a 1.5-fold decrease. DEGs were subjected to gene ontology analysis using the Database for Annotation, Visualization and Integrated Discovery (DAVID: https://davidbioinformatics.nih.gov/home.jsp).

### Statistics

A Student’s *t* test or one-way analysis of variance (ANOVA) was used to analyze differences in mean values between groups. All results are expressed as means ± standard errors of the means (SEM). *P* values of ≤0.05 were considered statistically significant (*, *P* ≤ 0.05; **, *P* ≤ 0.01; ***, *P* ≤ 0.001; ****, *P* ≤ 0.0001; n.s., not significant). “ns” stands for data with *P* value >0.05 and hence is not significant. Fold changes are designated by a number followed by X above the significance values.

## Data Availability

All the RNAseq reads data will be deposited in NCBI (Bioproject ID: PRJNA1139810; Biosample ID: SAMN42785804 [DMSO] and SAMN42786772 [PARP14 inhibitor treatment]) and will be made public upon acceptance. All other data is now available at the following link: 10.6084/m9.figshare.c.7766243.

## SUPPLEMENTAL MATERIAL

Supplemental Material mbio.02299-25-s0001.docx Figures S1-S8 and Tables S1-S3.

RNA-Seq Data mbio.02299-25-s0002.xlsx All RNA-Seq data comparing DMSO- and PARP14i- treated cells.

ASM does not own the copyrights to Supplemental Material that may be linked to, or accessed through, an article. The authors have granted ASM a non-exclusive, world-wide license to publish the Supplemental Material files. Please contact the corresponding author directly for reuse.
