# Supplementary material for: PARP14 is an interferon-induced host factor that promotes IFN production and affects the replication of multiple viruses
Source: mBio. 2025 Sep 12;16(10):e02299-25. doi: 10.1128/mbio.02299-25 (PMC12505956; doi:10.1128/mbio.02299-25)
Supplement: Supplemental Material — Figures S1-S8 and Tables S1-S3. [file mbio.02299-25-s0001.docx]

**Supplementary Information**

**PARP14 is an interferon (IFN)-induced host factor that promotes IFN production and affects the replication of multiple viruses**

Srivatsan Parthasarathy^1^, Pradtahna Saenjamsai^1^, Hongping Hao^1^, Anna Ferkul^1^, Jessica J. Pfannenstiel^1^, Daniel S. Bejan^2^, Yating Chen^3^, Ellen L. Suder^4,5,6^, Nancy Schwarting^1^, Masanori Aikawa^7,8,9^, Elke Muhlberger^4,5,6^, Adam J. Hume^4,5,6^, Robin C. Orozco^1^, Christopher S. Sullivan^3^, Michael, S. Cohen^2^, David J. Davido^1^, Anthony R. Fehr^1*^

*^1^Department of Molecular Biosciences, University of Kansas, Lawrence, Kansas 66045, USA*

*^2^Department of Chemical Physiology and Biochemistry, Oregon Health Sciences University, Portland, OR, 97239, USA*

*^3^Department of Molecular Biosciences, University of Texas, Austin, TX, 78712, USA*

*^4^Department of Microbiology, Boston University School of Medicine, Boston, MA, 02118, USA*

*^5^National Emerging Infectious Diseases Laboratories, Boston University, Boston, MA, 02118, USA*

*^6^Center for Emerging Infectious Diseases Policy & Research, Boston University, Boston, MA, 02118, USA*

*^7^Center for Excellence in Vascular Biology (P.K.J., M.A., E.A.), Brigham and Women's Hospital, Harvard Medical School, Boston, MA, 02115, USA.*

*^8^Center for Interdisciplinary Cardiovascular Sciences (M.A., E.A.), Brigham and Women's Hospital, Harvard Medical School, Boston, MA, 02115, USA.*

*^9^Channing Division of Network Medicine (M.A.), Brigham and Women's Hospital, Harvard Medical School, Boston, MA, 02115, USA.*

*Corresponding author: Dr. Anthony R. Fehr, Molecular Biosciences, University of Kansas, Lawrence, Kansas, USA, Tele: 785-864-6626

**CONTENTS**

**FIGURES S1-S8**

**TABLES S1-S3**

**SUPPLEMENTAL FIGURES**

**Fig. S1. PARP14 promotes IFN production following poly(I:C) treatment.** A) WT and PARP14 KO clone 23 (C23) A549 cells were transfected with 0.5μg/mL poly(I:C), at 18 hpt RNA was isolated from cells and the level of IFNβ mRNA was quantified using qPCR using ΔCt method. Data shown in D are from 1 experiment representative of 3 independent experiments with N=3 for each experiment. B) WT and PARP14 KO NHDF cells were transfected with 0.5μg/mL poly(I:C), at 18 hpt RNA was isolated from cells and the level of IFN-β mRNA was quantified using qPCR using ΔCt method. Data shown in A and B are from 1 experiment representative of 3 independent experiments with N=3 for each experiment. C) 293T cells were mock transfected (M) or transfected with plasmid expressing GFP-PARP14 G832E. At 24 hpt, cells were treated with PARPi for 4 hrs. Cells were then collected and analyzed by immunoblot with indicated antibodies. Data shown in B are from 1 experiment representative of 2 independent experiments. D) A549 WT cells were treated with either DMSO, negative control (CTL) or PARP14 Degron (DEG) for 6 hrs and then wither mock transfected or transfected with 0.5μg/mL of poly(I:C) with the corresponding treatments. At 18 hpt cell lysates were collected and PARP14 protein levels were determined by immunoblotting using β-actin as a loading control. E) WT A549 cells transfected with poly(I:C) were treated with 1 μM PARP14 Degron control (CTL) or PARP14 Degron (DEG) for 18 hours. RNA was isolated from cells at 18 hpt and IFN-β mRNA was quantified by qPCR using ΔCt method. Data shown in E are from 1 experiment and are representative of 3 independent experiments with N=3 for each experiment. F) A549 PARP14KO cells were transfected with 0.5μg of YFP-PARP14 overexpression plasmid (WT and Mac1 catalytic mutant), lysates were collected 48 hours post transfection and analyzed for PARP14 and GAPDH through western blot. Statistics in A, B, and E were determined using a non-parametric t-test.

**Fig. S2. PARP14 promotes IFN production following Mac1 mutant CoV infection.** A) Floxed Parp14 *cre*- (*Parp14+/+*) and *cre*+ (*Parp14-/-*) BMDMs were treated with 4-OHT and 24 hours later were infected with MHV WT and N1347A at an MOI of 0.1. At 12hpi, cell lysates were harvested and levels of PARP14 and β-actin were determined by immunoblotting. B) RNA was isolated from BMDMs and DCs and CXCR1 mRNA levels were quantified using qPCR using ΔCt method. Data shown in B are the combined data from 2 independent experiments with N=6 biological replicates for each group. Statistics were determined using a standard t-test. C) *Parp14+/+* and *Parp14-/-* BMDMs were infected with WT and N1347A MHV-JHM at an MOI of 0.1. RNA was collected 12 hpi and ISG15 mRNA levels were quantified using qPCR using ΔCt method. Data shown in C are from 1 experiment and are representative of 3 independent experiments with N=3 biological replicates for each experiment. Statistical analysis was done using a one-way ANOVA.

**
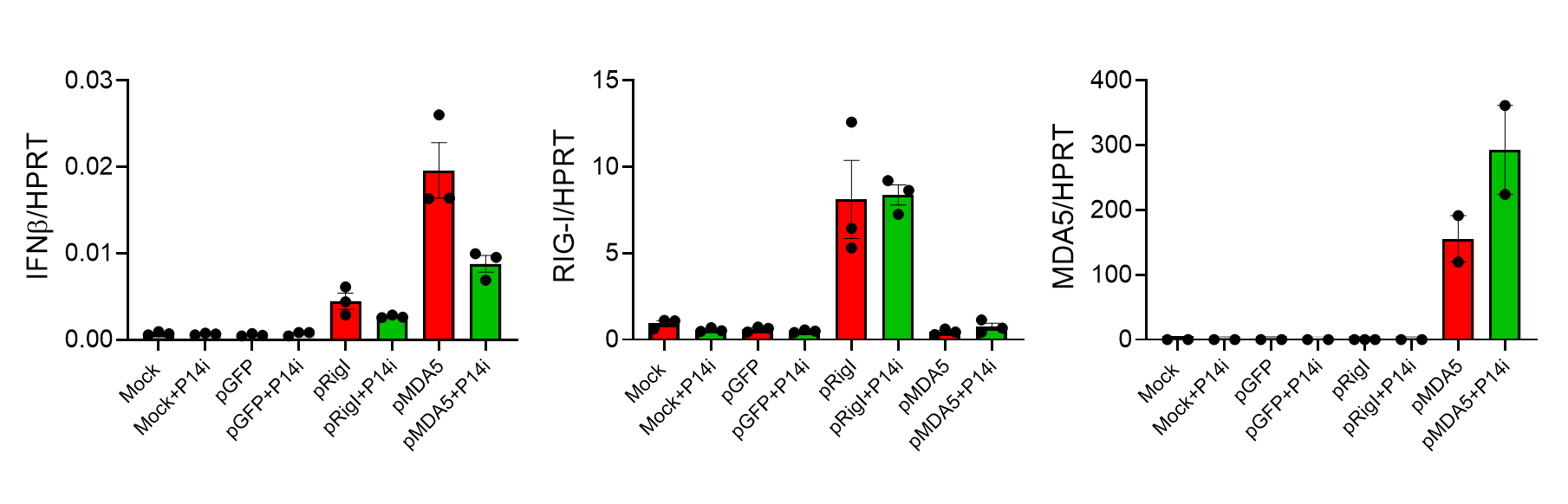
Fig. S3.** **PARP14 promotes MDA-5 dependent IFN-𝛽 production.** A549 cells were transfected with no plasmid (mock), pcDNA-GFP, pRIG-I and pMDA5 for 48 hours and IFN-β, RIG-I, and MDA5 mRNA levels were quantified by qPCR using ΔCt method with N=3 biological replicates for each group.

**Fig S4. PARP14 inhibits the replication of Mac1-mutant MHV and SARS-CoV-2.** A) BMDMs and B) DCs were infected with WT and N1347A MHV at an MOI of 0.1 and then treated with DMSO or PARP14i (1 𝜇M). At 20hpi cells and supernatants were collected and progeny virus was quantified by plaque assay. C) WT A549-ACE2 cells (without IFN𝛾 treatment) were infected with WT (red bars) and 𝛥Mac1 (green bars) SARS-COV-2 at an MOI of 0.1 and treated with DMSO or PARP14i (1 𝜇M) at 1 hpi. At 48 hpi cells and supernatants were collected and progeny virus was quantified by plaque assay. Data shown are from 1 experiment and are representative of 3 independent experiments with N=3 biological replicates for each experiment. Statistical analysis for A and B was done using standard t-test and statistical analysis for C was done using one-way ANOVA.

**Fig S5.** **PARP14 restricts the replication of HSV-1 in A549 cells.** WT and PARP14 KO A549 cells were infected with HSV-1 at an MOI of 0.01 PFU/cell. Cells and supernatants were collected at indicated time points and progeny virus was quantified by plaque assay on Vero cells. N=3 biological replicates per group at each timepoint. Statistical analysis was done using a one-way ANOVA.

**Fig S6.** **PARP14 does not impact LCMV infection.** A) Gating strategy used to identify LCMV^cl13^(+) cells by flow cytometry. B) WT and PARP14 KO BMDMs were infected with LCMV^cl13^ at an MOI of 1 and the % of LCMV^cl13^ (+) cells were quantified by flow cytometry. These data are from 1 experiment representative of 3 independent experiments, N=3 per group.

**Fig S7. Generation of PARP14 knockout (KO) A549-ACE2 cell lines using CRISPR/Cas9.** A549-ACE cells were transfected with 3 plasmids encoding small guide RNAs targeting PARP14, Cas9, and GFP (See Methods). The highest GFP expressing cells were isolated as single cell clones and tested by western blot for presence of PARP14.

**Fig S8. Reduced VSV replication in A549 PARP14 KO cells is not due to IFN.** A) A549 WT and PARP14 KO cells were infected with VSV-GFP at an MOI of 1 and treated with 1 μM PARP14 inhibitor (PARP14i). RNA was isolated from cells at 10 hpi and IFN-𝛽 mRNA was quantified by qPCR using ΔCt method. Data shown in A are from 1 experiment and are representative of 2 independent experiments with N=3 biological replicates for each experiment. B) A549 WT and PARP14 KO cells were infected with VSV-GFP at an MOI of 1 and treated with 1 μM JAK inhibitor (JAKi). RNA was isolated from cells at 10 hpi and PARP14 mRNA was quantified by qPCR using ΔCt method. Data shown in B are from 1 experiment and are representative of 2 independent experiments with N=3 biological replicates for each experiment. C) A549 WT and PARP14 KO cells were infected with VSV-GFP at an MOI of 1 and treated with 1 μM JAK inhibitor (JAKi). At 10 hpi, cells and supernatants were collected, and the progeny virus was quantified by TCID_50_. Results in C are from one experiment representative of three independent experiments with N=3 biological replicates for each experiment. Statistical analysis was done using a one-way ANOVA.

**SUPPLEMENTAL TABLES**

**Table S1. Guide RNAs for creation of PARP14 KO mice**

**Table S2. PARP14 KO genotyping primers**

**Table S3. qPCR primers**
